# The Use of Dapagliflozin in the Treatment of Children With Severe Insulin Resistance

**DOI:** 10.1155/pedi/6859764

**Published:** 2025-12-20

**Authors:** Najyya Attia, Khalid Al Noaim, Manal Mustafa, Suliman H. Al Fifi, Ibrahim Al Alwan, Nandu Thalange, Amir Babiker

**Affiliations:** ^1^ College of Medicine, King Saud Bin Abdulaziz University for Health Sciences, Jeddah, Saudi Arabia, ksau-hs.edu.sa; ^2^ Division of Pediatric Endocrinology, King Abdullah Specialized Children Hospital, King Abdulaziz Medical City, Jeddah, Saudi Arabia, ngha.med.sa; ^3^ Research Office, King Abdullah International Medical Research Centre, National Guard Health Affairs, Jeddah, Saudi Arabia, ngha.med.sa; ^4^ Department of Pediatrics, College of Medicine, King Faisal University, Al-Ahsa, Saudi Arabia, kfu.edu.sa; ^5^ Division of Pediatric Endocrinology, Al Jalila Children’s Speciality Hospital, Dubai, UAE, aljalilachildrens.ae; ^6^ Department of Child Health, College of Medicine, King Khalid University, Abha, Saudi Arabia, kku.edu.sa; ^7^ College of Medicine, King Saud Bin Abdul-Aziz University for Health Sciences, Riyadh, Saudi Arabia, ksau-hs.edu.sa; ^8^ Division of Pediatric Endocrinology, King Abdullah Specialized Children Hospital, King Abdulaziz Medical City, Riyadh, Saudi Arabia, ngha.med.sa; ^9^ Research Office, King Abdullah International Medical Research Centre, National Guard Health Affairs, Riyadh, Saudi Arabia, ngha.med.sa

**Keywords:** dapagliflozin, insulin resistance type 1A, Rabson–Mendenhall syndrome, subcutaneous insulin resistance, type 1 diabetes mellitus

## Abstract

**Background:**

Managing severe insulin resistance (IR) is challenging, necessitating a multifaceted approach, including dietary restriction, exercise, and pharmacotherapy. This paper will detail our utilization of dapagliflozin in a series of cases involving patients with severe IR of various etiology and inadequate glycemic control.

**Case Studies:**

We describe six cases of extreme IR with distinct clinical diagnoses: four with Rabson–Mendenhall syndrome (RMS), one with IR type 1A, and a patient with type 1 diabetes mellitus (T1DM) and severe subcutaneous (SC) IR. These cases exhibit the observable characteristics of IR, characterized by an inability to effectively manage blood glucose (BG) with a standard treatment plan. Every case had a remarkable response to dapagliflozin. Subsequent assessment demonstrated improved HgbA1C, fasting glucose, insulin, and C‐peptide concentrations. Furthermore, several cases demonstrated improvement in the clinical manifestations of IR following the administration of dapagliflozin, while others showed a reduction in the frequency of diabetic ketoacidosis (DKA). There were no documented adverse reactions with the use of dapagliflozin for a duration of 2–4 years in these patients.

**Conclusion:**

Dapagliflozin appeared both safe and effective as a standalone treatment or when used alongside other antidiabetes medications such as insulin in a case series of children with T1DM and severe IR or IR syndromes (IRS).

## 1. Introduction

Insulin resistance (IR) is defined as the inability to achieve a normal physiological plasma glucose disposal rate at the normal physiological plasma insulin level; hence, it results in an increased fasting plasma insulin level [1]. IR could be primary or secondary [2]. Primary causes lead to a group of extreme IR syndromes (IRS) such as leprechaunism, Rabson–Mendenhall syndrome (RMS), and type A IRS [3]. In these rare encounters, management is challenging depending on the type of genetic defect, and extremely high doses of insulin might be required [4]. On the other hand, acquired IR has a milder phenotype. Acquired causes include physiological states, such as puberty and pregnancy, or pathological disorders such as obesity, cirrhosis, uremia, stress, and endocrine disorders such as acromegaly, thyrotoxicosis, insulinoma, glucagonoma, cushing’s syndrome, pheochromocytoma, and autoantibodies against insulin receptors (INSRs) [5].

Patients with type 1 diabetes mellitus (T1DM) might develop IR due to several factors, such as genetic or familial IR, acquired lifestyle IR, or an uncommon condition of subcutaneous (SC) IR that is very challenging to manage [6–8]. Interestingly, IR in T1DM might also be related to the route of administration of therapeutic exogenous insulin. Insulin absorbed from the SC injection site results in relative peripheral hyperinsulinemia and hepatic hypoinsulinemia compared with normal physiology [8]. Chronic adaptation to this combination could reduce peripheral insulin‐mediated glucose uptake and increase hepatic glucose production [9].

Treatment of severe IRS is challenging, with different strategies being employed, including carbohydrate restriction, exercise, and drug therapy. This includes high‐dose insulin therapy, metformin, and IGF‐1 analogs [4]. Research on newer medications is required, given the significant challenges encountered in managing these patients [10, 11]. The sodium‐glucose‐linked transporter 2 (SGLT2) inhibitor, dapagliflozin, is a medication used to treat T2DM with a good safety profile in pediatric patients [12]. Several reports have observed beneficial effects on insulin sensitivity and glycemic control in insulin‐resistant patients [4, 13]. Dapagliflozin controls blood glucose (BG), independent of insulin action or secretion, through a selective inhibition of SGLT2 located in the proximal tubule of the kidney [14]. This results in increased glucose disposal by increasing urinary glucose excretion [14].

In this article, we present our experience of using dapagliflozin in pediatric patients with severe IR and poor glycemic control. All had significantly improved glycemic control following introduction of dapagliflozin.

## 2. Case Series

We report on six cases with different types of IR that responded well to dapagliflozin as an add‐on therapy, after periods of struggling with the control of hyperglycemia using insulin alone or other antidiabetes medications, without complications (Table 1).

**Table 1:** Summary of the clinical, laboratory, and management data in the reported cases.

Clinical/laboratory data	Note/reference	Case 1	Case 2	Case 3	Case 4	Case 5	Case 6
Diagnosis	—	IR syndrome type A	T1D with SC IR	RMS	RMS	RMS	RMS
Genetic result	—	Homozygous mutation in R141W in *INSR* gene	None	*INSR* gene mutation	*INSR* gene mutation	*INSR* gene mutation	*INSR* gene mutation
Age at initial treatment	Years	12	11	13	6	7	10
Starting dose	Once daily	5 mg	2.5 mg	5 mg	5 mg	5 mg	5 mg
Maximum dose	Once daily	10 mg	5 mg	5 mg	5 mg	5 mg	5 mg
Duration of treatment	Years	3	2.5	4	3	2	6
HgbA1c	Before dapa (3.9%–5.7%)	13%	14%–15%	8.5%	6.6%	6.3%	10%
HgbA1C	After dapa (3.9%–5.7%)	9%	8%	6.2%	5.8%	6%	7.9%
Weight centiles	Before dapa	25th	5th–10th	<3^rd^	3^rd^–10th	3^rd^–10th	50th
Weight centiles	After dapa	50th–75th	10th–25th	>3^rd^	10th–25th	3^rd^	50th–75th
Fasting insulin level	2.2–49.6 uU/mL	160.0	ND	>600.0	181.7	349.4	385.0
C‐peptide	257–2241 pmol/L	1860	200	4416	620	1752	2063
Anti‐GAD Ab	—	Negative	Positive	Negative	Negative	ND	Negative
Anti‐insulin Ab	—	Negative	Positive	Negative	ND	ND	ND
Anti‐IC Ab	—	Negative	Positive	Negative	ND	Negative	Negative
IGF‐1	Variable per age group^a^	191	ND	84	74	90	ND
LDL‐C	<2.8 mmol/L	1.56	2.59	1.74	1.80	2.27	1.92

Abbreviations: Ab, antibodies; Dapa, dapagliflozin; GAD, glutamate decarboxylic acid; HgbA1c, glycated hemoglobin; IC, Islet cell; IGF‐1, insulin like growth factor; INSR, insulin receptor; LDL, low‐density lipoprotein; ND, not done; RMH, Rabson–Mendenhall syndrome; SC IR, subcutaneous insulin resistance.

^a^All reported IGF‐1 levels in these patients were within the normal range.

### 2.1. Case 1

**Figure 1:**
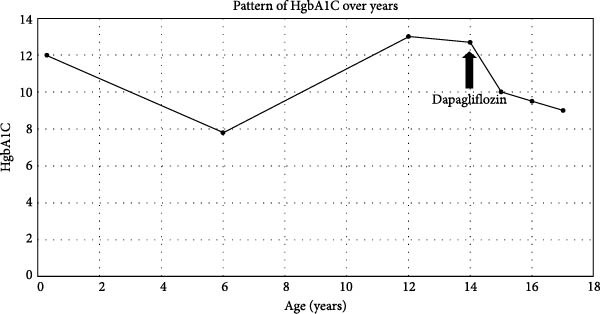
A flowchart of HbA1C level in Case 1 over years of treatment.

A 16‐year‐old girl with severe congenital IR type A was genetically confirmed with homozygous mutation in R141W in the *INSR* gene. She is the first child of nonconsanguineous parents. She has three other healthy sisters. One of her mother’s cousins has RMS on rhIGF1 (Increlex). She first presented at the age of 50 days with hyperglycemia (750 mg/dL) and metabolic acidosis requiring high‐dose insulin infusion therapy (2 units/kg/h). Her labs showed high insulin and C‐peptide levels. She started initially on an insulin pump for 2 months, before switching to rosiglitazone alone once daily. Her BG levels were still suboptimal; hence, she started increlex at the age of 6 years with partial improvement. At the age of 12 years, she developed a second episode of ketoacidosis again requiring high‐dose intravenous (IV) insulin infusion. Thereafter, the patient was started on SC insulin multidose injections at 8 units/kg/day before switching to continuous subcutaneous insulin infusion (CSII) therapy. However, she continued to have poor glycemic control, with HbA1C persistently exceeding 12%. Over the last 3 years, the patient experienced a total of six episodes of diabetic ketoacidosis (DKA), four of which were severe and occurred in the year prior to starting dapagliflozin. The last two episodes happened just before initiating the treatment. The first episode was triggered by cellulitis and metabolic acidosis, requiring high doses of IV insulin (600–800 IU/day) for less than 24 h; she was discharged after 1 week when her hyperglycemia stabilized. The second episode occurred just before the initiation of dapagliflozin and was caused by acute gastroenteritis. Following this episode, she was prescribed Increlex and continued on an insulin pump while trialing dapagliflozin. However, persistent high blood sugar levels and severe skin reactions led to the discontinuation of pump therapy after 1 week, transitioning her back to SC insulin. Since starting dapagliflozin, the patient has experienced two mild to moderate DKA episodes within the first year, both triggered by intercurrent infections and resolved quickly with IV insulin. Additionally, she required an increase in basal insulin doses due to weight gain associated with advancing puberty. With the trial of once‐daily oral dapagliflozin (5 mg), the patient showed a notable improvement in her glycemic control and her HbA1C dropped for the first time to <10% over 1 year period (Figure 1). The dose of dapagliflozin was subsequently increased to 10 mg once daily oral dose for better glycemic control. She continued on dapagliflozin 10 mg daily, increlex 40 units twice a day, and CSII with concentrated insulin (Humulin R U‐500) with a further drop in her A1c over the subsequent 2 years period (Figure 1). Her total insulin level also dropped from 219 to 105 uU/mL over a 10 months period. A recent exam showed severe acanthosis nigricans around the neck, in the axillae and groin area on both sides, as well as generalized hirsutism and hypertrophy where the insulin pump was inserted. She was on Tanner stage 5 in puberty. The last lab results showed HbA1C of 9.5% with a normal IGF‐1 level, liver profile, and lipid profile. Since she started on dapagliflozin besides concentrated insulin via pump, her DKA has become much less frequent than previously.

She is under regular follow‐up with the psychologist in her local hospital and is currently on the following medications: an insulin pump with a concentrated insulin (Humulin R U‐500), increlex 40 units SC twice daily, and dapagliflozin 10 mg orally once daily without side effects from medications for the last 4 years.

### 2.2. Case 2

A 14‐year‐old girl presented in DKA at the age of 10 years. T1DM was confirmed by positive autoantibodies (GADA, ICA, IAA). She responded well to DKA management, but it was difficult to achieve effective glycemic control prior to discharge. A year later, she still had an extremely high HbA1C ranging between 13% and 15%, despite an insulin dose of 2.7 units/kg/day. The issue of adherence was raised, but the mother confirmed that she gave the insulin injections without missing doses, and the girl did not refuse the injections. To confirm this, the patient was electively admitted for supervised insulin treatment using an “imprisonment tactic.” SC insulin was administered by the primary nurse to exclude the issue of adherence. She was then started on an IV insulin infusion after we confirmed the very high insulin requirement with different SC insulin regimens during this admission to evaluate the response of the body to IV insulin compared to the SC route. Surprisingly, she showed a significant reduction in insulin requirement via the IV route to control her hyperglycemia (less than 1 unit/kg/day of IV insulin within 2–3 h of treatment) (Table 2). However, her hyperglycemia returned when she switched back to the SC route at a dose of 2 units/kg/day, confirming SC IR. The primary team tried pump therapy with a total daily dose of around 2.5 units/kg/day with minimal improvement in her HbA1C, which was persistently above 12%. We therefore started a trial of dapagliflozin 5 mg orally once daily, with significant improvement in her glycemic control within 3 months as her HbA1c dropped to <9% and the insulin dose dropped to 1.6 units/kg/day. She completed 4 years without significant complications and tolerated even an additional growth hormone therapy, with no DKA episodes.

**Table 2:** Serial blood glucose measurements following intravenous insulin infusion during hospital admission in Case 2.

Time (mins)	0 min	30 min	60 min	120 min	180 min^a^
Blood glucose level (mg/dL)	320	270	170	120	90

^a^Insulin infusion reduced from 0.1 to 0.05 unit/h at 180 min then stopped.

### 2.3. Cases 3 and 4 (Family 1)

#### 2.3.1. Case 3

A 13‐year‐old girl presented at the age of 9 years with polyuria and polydipsia, and extensive acanthosis nigricans in her neck, axilla, and groins. There were no symptoms of diabetes. Her parents were consanguineous, and her sister shared the same phenotype (Figure 2A). On examination, she had short stature and failure to thrive with height and weight below the 3rd centile. She had coarse facial features, hypertrichosis, skin tags, breast tanner stage 3, and clitoromegaly. Investigations showed extremely high insulin and C‐peptide levels, as well as high fasting and 2 h postoral tolerance test serum glucose levels of 8.5 mmol/L and 28.5 mmol/L, respectively. A genetic test revealed a variant of unknown significance (VUS) in the *INSR* gene. However, her phenotype was consistent with RMS. The patient was given oral dapagliflozin 5 mg once a day as a trial. Within 8 months of starting the medication, her HbA1C dropped from 8.5% to 6.2%, her fasting glucose dropped from 8.5 to 3.2 mmol/L, and her C‐peptide dropped from 4416 pmol/L to 1377 pmol/L (Table 3). She continued on dapagliflozin 5 mg orally once daily with good glycemic control, without any significant complications or DKA episodes.

Figure 2(A) The family pedigree of Case 3 and Case 4. (B) Signs of acanthosis nigricans around the umbilicus and behind the neck and in her knees and the axillae in Case 3. (C) Improvement in the darkening of the skin around the umbilicus 8 months after starting treatment in Case 3.

**Figure:**
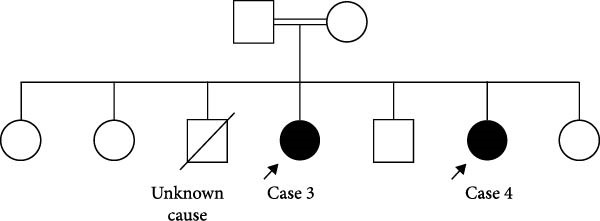
(A)

**Figure:**
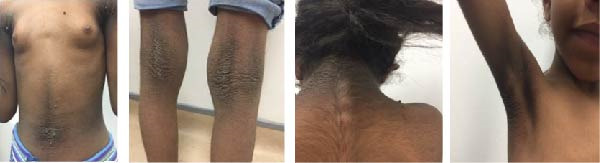
(B)

**Figure:**
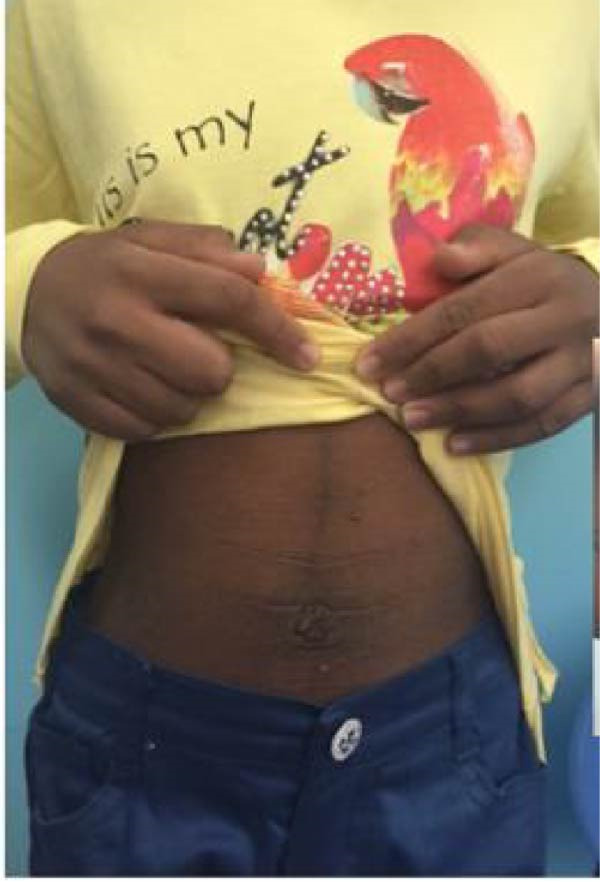
(C)

**Table 3:** Changes in metabolic markers of dysglycemia in patients 3–6 with insulin resistant syndromes: Measurements pre‐dapagliflozin (baseline) and post‐dapagliflozin (2–12 months after treatment).

Investigations		Case 3			Case 4			Case 5			Case 6		
Lab test	Reference	Pre	2–6 months	7–12 months	Pre	2–6 months	7–12 months	Pre	2–6 months	7–12 months	Pre	2–6 months	7–12 months
Insulin level	2.2–49.6 uUmL	543.0	299.1	131.5	595.6	253.4	159.2	349.4	175.7	101.8	436.7	192.5	134.6
HgbA1c	3.9–5.7%	8.5	6.2	7.1	5.9	5.8	6.0	6.3	5.7	6.0	10.0	9.0	7.9
C‐peptide	257–2241 pmol/L	4416	1377	653	620	442	ND	601	1752	760	2063	1543	768
Fasting BG	3.3–5.6 mmol/L	8.5	3.2	NA	3.5	3.3	ND	3.7	5.4	3.3	22	7.2	5.5

Abbreviations: BG, blood glucose; HgbA1c, glycated hemoglobin; ND, not done.

Furthermore, her acanthosis of the skin in the axilla, groin, and neck regions may have subsided or shown significant improvement (Figure 2B,C). This positive response suggests that the treatment approach had a beneficial impact on her condition.

#### 2.3.2. Case 4

A 9‐year‐old girl, the sibling of Case 3, came to our clinic at the age of 6 years with a 1‐year history of progressive darkening of the skin in the axilla, groin, and neck region. Unlike her sister, she did not experience symptoms of polyuria or polydipsia. Her development was noted to be normal. There was a family history of T2DM in the grandparents during old age, as well as consanguinity (Figure 2A).

**Figure 3:**
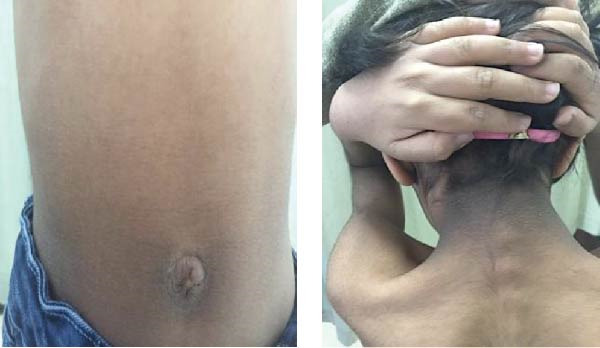
Acanthosis nigricans behind the neck and around the umbilicus of Case 4.

Her HgbA1c was 6.6%. An oral glucose tolerance test showed a fasting BG level of 98 mg/dL and a 2‐h BG level of 210 mg/dL, confirming impaired glucose tolerance or early‐stage diabetes. On examination, the patient was slim, with weight just above the 3rd percentile. She had severe acanthosis nigricans in her neck, axilla, and around the umbilicus, and her skin was notably dry (Figure 3). Based on these results, as well as the clinical presentation of progressive darkening of the skin and her sister’s similar condition, the decision to start dapagliflozin was made. The same *INSR* gene mutation as her sister was found. HgbA1c levels and other metabolic markers improved after initiating treatment with dapagliflozin (Table 3).

### 2.4. Cases 5 and 6 (Family 2)

#### 2.4.1. Case 5

A 9‐year‐old boy initially presented at the age of 7 years with progressive and extensive acanthosis nigricans observed in the neck, axilla, and groin regions (Figure 4). Surprisingly, there were no symptoms of diabetes. Parents were consanguineous. His two older sisters had been diagnosed with T1DM treated with insulin, and an older sister was diagnosed with T2DM and progressive acanthosis nigricans (Case 6) (Figure 4). He was underweight (weight −3.15 SDS) and short (height ‐2.5 SDS). An oral glucose tolerance test showed normal fasting glucose, but his 2‐h value was 14.3 mmol/L (258 mg/dL). His HbA1c was 6.3%. Islet‐cell antibodies were negative. A genetic study showed a pathological mutation in *INSR* gene consistent with RMS.

Figure 4(A) The family pedigree of Case 5 and Case 6. (B) Signs of acanthosis nigricans around the neck and in axillae in Case 5. (C) Signs of acanthosis nigricans around the umbilicus and behind the knee in Case 6.

**Figure:**
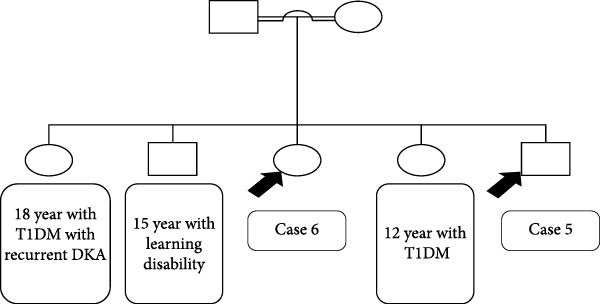
(A)

**Figure:**
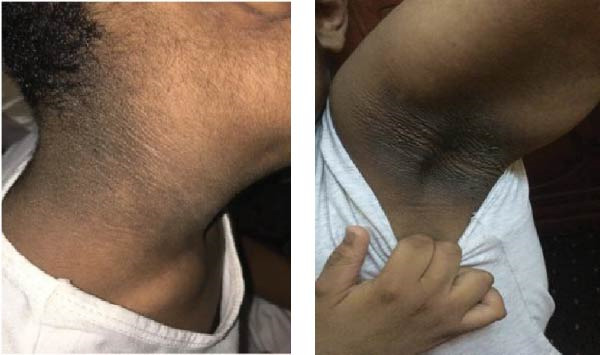
(B)

**Figure:**
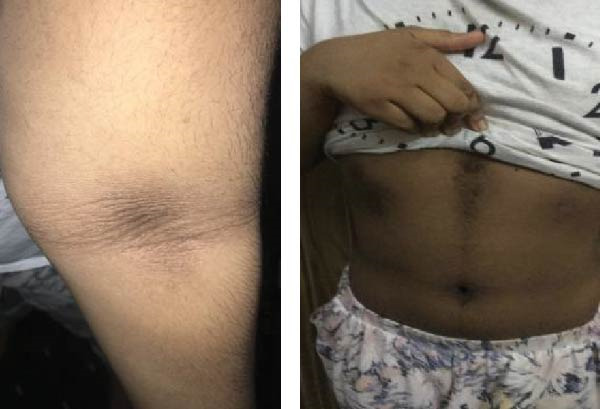
(C)

Following the diagnosis, the patient was prescribed dapagliflozin at a dose of 5 mg orally once daily. Subsequent laboratory tests demonstrated improvements in HbA1c levels, C‐peptide levels, fasting insulin levels, and fasting glucose levels (Table 3). Concurrently, there was notable clinical improvement, with an increase in weight, improvement in acanthosis nigricans, and stable BG readings within the range 90–130 mg/dL.

#### 2.4.2. Case 6

The patient was initially diagnosed with T2DM at the age of 10 years, when she presented with polyuria, polydipsia, and acanthoses nigricans (Figure 4). Her initial BG level was 22 mmol/L (396 mg/dL). She was started initially on metformin, but her BG was not controlled. After that, she was switched to insulin therapy. At age 15 years, her younger brother (Case 5) was diagnosed with RMS. Examination showed normal weight of 50%, with severe acanthosis of the neck, axilla, and groin, with dry skin.

Due to her severe progressive acanthoses nigricans and uncontrolled T2DM, she was admitted to the hospital to start dapagliflozin 5 mg once daily orally under observation. During the admission, insulin doses were stopped, and BG levels were maintained in the range 90–130 mg/dL. Dapagliflozin treatment led to marked improvements in HgbA1C, insulin, and C‐peptide levels (Table 3). Her genetic study confirmed the same pathologic mutation in *INSR* as her brother.

## 3. Discussion

Treatment of severe IR is challenging. Different strategies were implemented, such as diet restriction, exercise, and oral hypoglycemics [4]. A short period of extensive caloric intake restriction could not improve the sensitivity to exogenous insulin therapy [15]. While regular exercise may improve the glycemic control of patients with T2DM, it is not effective for severe SC IR [15]. Drug therapy for severe insulin syndromes is currently disappointing. High insulin doses frequently fail to achieve good glycemic control, particularly in cases of severe resistance syndromes [3]. Medications that stimulate endogenous insulin production have a poor effect on glycemic control because these patients already have high endogenous insulin levels [15]. Generally, insulin and IGF‐1 receptors share more than 50% of the sequence features and more than 80% of the intracellular kinase domain. Both insulin and IGF‐1 can stimulate each other’s receptors [16]. Therefore, rhIGF‐1 may be used to treat severe IRS. It can improve glycemic control in short‐term observational studies, but the efficacy and safety of long‐term therapy are unclear [17]. Other medications that work by increasing insulin sensitivity, such as metformin and in one case, rosiglitazone, were unsuccessful [15].

Dapagliflozin promotes glucosuria, reduces glucotoxicity, and enhances tissue insulin sensitivity by restoring insulin signaling pathways in peripheral tissues, particularly in skeletal muscle. This leads to improved insulin‐mediated glucose disposal and overall sensitivity [18]. By lowering plasma glucose levels, dapagliflozin decreases the need for high insulin doses, which helps reduce IR. In cases of severe IR, managing hyperglycemia alleviates secondary metabolic stresses that worsen the condition [19, 20]. These effects may also lower the risk of DKA by improving glycemic stability, provided that insulin is not excessively reduced. Over‐reduction of insulin can paradoxically increase the risk of euglycemic DKA with SGLT2 inhibitors due to elevated glucagon levels and a shift towards lipolysis and ketogenesis [21]. Therefore, careful patient selection and monitoring are essential. The efficacy of dapagliflozin depends on renal function; therefore, its efficacy is reduced in patients with moderate or severe renal impairment [22, 23]. Some RMS cases have renal abnormalities; thus, it is recommended to screen patients for kidney disease before starting dapagliflozin [24, 25]. The safety and efficacy of dapagliflozin have been studied in children and adolescents with T2DM, where it was found to be well tolerated and showed equivalently controlled diabetes as in adults [12]. It also reduces weight and the frequency of hypoglycemia compared to metformin for patients with T2DM [26].

SC IR and IRS differ significantly in severity and underlying mechanisms. Clinically, SC IR may present with localized symptoms like lipodystrophy or poor glycemic control, while IRS exhibit systemic issues, including developmental delays and significant health complications [27–31]. Management of SC IR focuses on improving insulin absorption, whereas IRS require comprehensive, multidisciplinary approaches to address underlying genetic or metabolic abnormalities [27–31]. Overall, while SC IR is generally manageable and less severe, IRS pose significant health challenges that necessitate complex treatment strategies. However, including Case 2, which involves a SC IR, a patient with T1DM demonstrates the efficacy of dapagliflozin in this less severe yet challenging IR condition [32]. Unlike more severe IRS, this SC IR may necessitate IV insulin administration, which is impractical for long‐term therapy. Highlighting the differences between these conditions underscores that while SC IR may not be as severe, it still requires effective treatment strategies, making the role of dapagliflozin particularly relevant in improving patient outcomes in these scenarios. Moreover, while insulin therapy is the cornerstone treatment for T1DM, glycemic control is suboptimal in more than 70% of these patients [26]. Increasing insulin doses might improve glycemic control, but it also, with unclear guidance of an effective dose, increases the risk of life‐threatening hypoglycemia [33]. Randomized control trials (DEPICT‐1 and DEPICT‐2) showed that adding dapagliflozin to the insulin therapy of adults with T1DM improved glycemic control without insulin‐related side effects [34, 35]. Interestingly, the risk of DKA was lower in people treated with dapagliflozin (5 mg/day) with a BMI ≥ 27 kg/m^2^ compared with others [36]. DKA is a significant risk in T1DM patients, and this risk is amplified by use of SGLT2 inhibitors. These drugs raise *β*‐hydroxybutyrate, the principal ketone in plasma. Further trials are needed as SGLT2 inhibitors are not routinely used in T1DM [37]. However, use in exceptional circumstances, such as in Case 2, above, may be justifiable if done cautiously and with close supervision and patient education. In August 2019, the National Institute for Health and Care Excellence (NICE) in the UK recommended using dapagliflozin as adjuvant therapy with insulin in adults with T1DM who had a BMI ≥ 27 kg/m^2^, but the United States Food and Drug Administration refused to approve its use due to concerns about euglycemic DKA [27], a recognized complication of SGLT2 inhibitors [38]. As of recent updates, dapagliflozin is no longer recommended for adults with T1DM in the UK. The recent recommendations from both the European Medicines Agency (EMA) and NICE regarding dapagliflozin are that they have been revoked due to concerns about an increased risk of DKA [38]. This situation leaves sotagliflozin as the only SGLT2 inhibitor approved by the EMA for use alongside insulin in the treatment of T1DM [39].

Progressive kidney disease is a serious long‐term complication of diabetes; however, dapagliflozin and other SGLT2 inhibitors have proven renoprotective benefits in adults [40, 41]. Recently, dapagliflozin was shown to reduce proteinuria in a small group of adolescent patients [42, 43]. Dapagliflozin was chosen over other SGLT2 inhibitors in this report due to its proven efficacy, favorable safety profile, and supportive literature for patients with severe IR [44–47]. It has demonstrated significant improvements in glycemic control and insulin sensitivity in T2DM and the safety profile is well‐established, particularly in pediatric populations, with lower adverse effects compared to other SGLT2 inhibitors [4, 13, 32, 48]. Existing studies indicate positive outcomes in IR cases, including enhanced HbA1c levels and reduced DKA episodes [47]. Additionally, dapagliflozin is one of the few SGLT2 inhibitors with specific pediatric studies, and its independent mechanism of action is beneficial for patients with severe IR, promoting glucose excretion without a high risk of hypoglycemia [44–47]. In addition, poor glycemic control in severe IRS contributes to adverse cardiovascular outcomes with increased mortality [46]. Dapagliflozin strikingly improved metabolic control, as shown in our cases, after long periods of glucose dysregulation with high insulin requirements.

Recent studies have highlighted the potential benefits of SGLT2 inhibitors in managing IRS, particularly in pediatric populations. For instance, recent studies have highlighted the successful use of SGLT2 inhibitors in challenging IR scenarios. For instance, a recent study reported on insulin‐resistant diabetes in RMS, which is relatively unresponsive to first‐line antidiabetic treatments, including metformin and insulin [48]. The study involved two patients with RMS treated with different sodium‐glucose cotransporter inhibitors: empagliflozin in an 11‐year‐old boy and dapagliflozin in a 12‐year‐old girl. In the first patient, empagliflozin was initiated at 2.5 mg/day and increased to 10 mg/day over 3 months [48]. During treatment, the time spent maintaining serum glucose within the target range increased by 2 h per day, with hemoglobin A1C dropping from >14% to 11.9%. However, due to insufficient further improvement with dose escalation, the dosage was reverted to 2.5 mg/day. In the second patient, dapagliflozin was started at 5 mg/day, resulting in a reduction of hemoglobin A1C from 8.5% to 6.2% after 6 months and insulin levels fell by over 50%. Both empagliflozin and dapagliflozin were well tolerated and improved glycemic control without significantly increasing ketonemia. This aligns with our findings, where dapagliflozin led to notable decreases in HbA1c levels across several cases, suggesting shared efficacy among SGLT2 inhibitors in similar contexts. In another report, they concluded that SGLT2 inhibitors appear to be a promising treatment option for INSR‐c, SIR, and possibly type‐A SIR in patients with stable diabetes [49]. However, the long‐term efficacy of SGLT2 inhibitors in these conditions requires additional research [49]. The comparative analysis of our case series with existing literature reveals that while SGLT2 inhibitors are increasingly recognized for managing IRS, the choice of specific agents can significantly influence treatment outcomes [48–50].

Dapagliflozin typically reduces total body weight slightly, mostly by lowering fat mass, visceral adipose tissue, and SC adipose tissue in patients with T2DM who are not adequately controlled with metformin [36]. However, most of our reported subjects showed an increase in weight percentiles after the dapagliflozin administration; they changed from growth failure to a normal percentile of weight for age and gender, reflecting a beneficial effect on insulin action.

## 4. Conclusion

The case series suggests that dapagliflozin holds a potential as a suitable treatment option for children and adolescents with severe IR. Dapagliflozin demonstrated both safety and efficacy when used as a monotherapy or in conjunction with other antidiabetes medications in our case series.

## Conflicts of Interest

The authors declare no conflicts of interest.

## Author Contributions

Najyya Attia and Khalid Al Noaim contributed equally to this work.

## Funding

No funding was received for this manuscript.

## Data Availability

The data that support the findings of this study are available from the corresponding author upon reasonable request.
